# Exploring the role of our contacts with pets in broadening concerns for animals, nature, and fellow humans: a representative study

**DOI:** 10.1038/s41598-023-43680-z

**Published:** 2023-10-10

**Authors:** Catherine E. Amiot, Christophe Gagné, Brock Bastian

**Affiliations:** 1 Département de Psychologie, Université du Québec à Montréal, Succ. Centre-Ville, C.P. 8888, Montreal, PQ H3C 3P8 Canada; 2 Melbourne School of Psychological Sciences, The University of Melbourne, 06, 05, Redmond Barry Building, Parkville, VIC 3010 Australia

**Keywords:** Psychology, Environmental social sciences

## Abstract

While pet ownership is normative in many occidental countries, whether humans’ proximal contacts with pets have implications for attitudes and behaviors toward other (non pet) animals, nature, and fellow humans, has received limited empirical attention. In a large representative sample, we investigate whether pet ownership and positive contact with pets are associated with more positive attitudes and heightened concerns for non-pet animals, nature, and human outgroups. A cross-sectional questionnaire survey was conducted among Canadian adults (619 pet owners, 450 non-pet owners). Pet owners reported more positive attitudes toward non-pet animals (e.g., wild, farm animals), higher identification with animals, more positive attitudes toward human outgroups, higher biospheric environmental concerns, higher human–environment interdependence beliefs, and lower usual meat consumption. Positive contact with pets was also associated with most of these outcomes. Solidarity with animals, a dimension of identification with animals, emerged as a particularly clear predictor of these outcomes and mediated the associations between positive contact with pets and positive attitudes toward non-pet animals, biospheric, egoistic, and altruistic environmental concerns, human–environment interdependence beliefs, and diet. Our results provide support for the capacity of pets to shape human consideration for a broad range of social issues, beyond the specific context of human-pet relations.

## Introduction

Human-pet relations are normative in many occidental countries, with a majority of people in the U.S., Canada, and Australia living with a least one pet^1–3^. Given their proximal nature, human-pet relationships can promote more positive attitudes and behaviors toward animals generally, including toward non-pet animals^4^. In this sense, pets can act as ‘ambassadors’ for other animals. The current study aims to explore this pets as ambassador effect more broadly, by examining whether pet ownership and contact with pets also have positive implications for our perceptions of human social groups, people’s concerns for the natural environment, and the extent to which they engage in common dietary habits such as meat-eating, a practice that impacts on both animals and the environment^5,6^. We also test the psychological processes through which these broadening effects take place, and which explain why contact with pets may lead to more positive and inclusive concerns for animals, nature, human outgroups, and lower meat consumption. To maximise the generalisability of the findings, these associations are tested in a large representative dataset.

### Extending the pets as ambassador hypothesis

According to the pets as ambassadors hypothesis^4^, contact with pets can serve as a springboard to more positive attitudes and moral considerations toward animals more generally (e.g., farm animals, wild animals). Empirical evidence has supported this contention, showing that pet owners tend to have more positive attitudes toward animals in general than non-pet owners^7,8^. In representative datasets, pet owners reported being involved in animal protection and in favor of awarding fundamental rights to animals to a greater extent then non-pet owners, but less accepting of eating animals (^9^, see also^10^). The quality of one’s relationship with a pet also plays a role: Reporting higher intensity of childhood pet relationships has been associated with more positive attitudes toward pets, more positive behaviors toward animals generally, and greater empathy toward humans (^11^, see also^12^). Stronger attachment to one’s pet was also related positively to ethical concern for non-pet animals and to lower levels of speciesism^13^. Yet, the pets as ambassadors effect could operate even more broadly, such that our contacts with pets may have implications not only for how we think about non-pet animals, but also for human outgroups, the natural environment, and for dietary habits which have repercussions for animal welfare and the environment.

**Figure 1:**
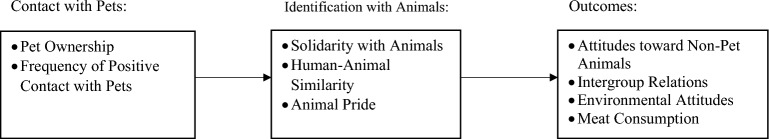
Mediation model tested.

Further, the specific psychological mechanisms through which the pets as ambassador effect operates remains under-investigated. One likely process through which contact with pets broadens our concerns, beyond the specific context of human-pet relations, pertains to identification with animals. Building on the intergroup relations literature^14,15^, identification with animals involves perceiving a psychological connection that ties and connects us to the other individuals within the group, namely, other animals (i.e., both human and non-human animals;^16^; see also^17^. Empirically, research on human-animal relations has shown that thinking about how animals are similar to humans increases people’s moral concerns for both non-human animals and for human outgroups, suggesting that thinking about how animals are similar to humans broadens our cognitive field and promotes a particularly inclusive recategorization process^18,19^. Building on this prior work, and as illustrated in Fig. 1, social identification with animals could represent a cognitive broadening/recategorization process which acts as a bridge between contact with pets (i.e., as an intergroup contact taking place between the members of different species;^20^) and heightened concerns toward different social groups.

Another reason for why contact with pets may broaden our concerns so that they extend beyond the specific context of our relations with pets pertains to the unique nature of the human-pet relationship. Human-pet relations are a particularly proximal and concrete type of human-animal relation, which can also be seen as prototypical of our relations with animals in general^21^. By caring and feeling affection for pets, and realising on a day-to-day basis the similarities we share with them (e.g., similar needs and learning processes; shared habitat), these relations may serve as a gateway for thinking more inclusively and developing broader social concerns^22^. On these bases, we argue that contact with pets may impact on a range of targets and social groups, and that this effect takes place by broadening our cognitive field and recategorizing ourselves into a particularly inclusive social identity, which includes all animals: human and non-human.

### Outcomes involving animals, humans, and the natural environment

To provide a test for this extended pets as ambassador hypothesis, the current study includes outcomes that concern animals, humans, and the natural environment. In addition to assessing people’s positive attitudes toward non-pet animals (i.e., farm and wild animals, pests), outcomes pertaining to *intergroup relations* involving human social groups will be tested, including participants’ attitudes toward relevant ingroups and outgroups, as well as their beliefs in the legitimacy of social hierarchies (i.e., social dominance orientation;^23^). A growing body of work reveals how human-animal relations offer a glimpse into human–human intergroup relations, how prejudice toward human groups is associated with prejudice toward animals (speciesism), and how these different types of prejudice share underlying ideological roots^24^. Herein, we specifically test if contacts with pets, as one specific type of human-animal relationship, has implications for human–human intergroup relations.

A further extension to the pets as ambassador effect is to examine whether it also extends to e*nvironmental attitudes.* Only a handful of studies to date have tested if pet ownership is associated with attitudes toward the environment, with mixed results; while pet ownership has been associated with pro-environmental attitudes in some studies^25,26^, in others, pet and non-pet owners did not differ in terms of their attitudes toward the environment^27,28^. Building on this emerging literature, the current study specifically focuses on people’s environmental concerns for animals and plants, humans, and themselves, as well as on their deeper feelings of connection and interdependence with animals and nature, as important indicators of environmental attitudes^29–34^.

If pets act as ambassadors for other non-pet animals, then it is also possible that this same effect may extend to people’s *meat consumption*. Prior studies investigating the association between pet ownership and meat consumption have mostly focused either on children or adolescent participants, or on adult participants asked to retrospectively report on their experience with pets in childhood (cf.^35^). For example, among adult participants, childhood attachment to one’s pet was associated with higher meat avoidance in adulthood^36^. Similarly, in a sample of university students, a positive association was uncovered between childhood pet involvement and ethical food avoidance practices such as vegetarianism (^11^; see also^12^). In a sample of children and adolescents, however, pet owners and non-owners did not differ in their levels of meat consumption^25^. While the tendency to morally prioritize humans over animals appears to develop with age through socialization processes^37^, focusing on the experience of pet ownership in adulthood and its possible associations with (lower) meat consumption could contribute to our understanding of the factors shaping a dietary behavior that has ethical and environmental implications. To this aim, the current study not only focuses on diet type and frequency of meat consumption, but also on people’s acceptance of different (e.g., normative) justifications for eating meat^38^.

## The current study

The current study contributes to the literature in two novel ways: First, we test and extend the pets as ambassadors hypothesis to include not only other animals, but also human social groups and nature. Given that human-pet relations can take different forms, the current study focuses on two types (i.e., indicators) of contact with pets, namely: pet ownership, as an objective and often intensive form of contact^39,40^, and the frequency of positive contact with pets, as a more subjective type of contact not strictly reserved to current pet owners^20,41^. Indeed, in occidental societies, it is rare to have absolutely no contact with pets^42^. Capturing this later type of contact, while less intensive, provides a strong test of the extended pets as ambassadors hypothesis. Specifically, we test if both pet ownership and positive contact with pets will be associated with more positive attitudes toward non-pet animals (i.e., farm animals, wild animals), higher pro-environmental concerns for animals, more positive attitudes toward human outgroups, and lower meat consumption. These propositions also align with intergroup contact research, which has shown that contacts with the members of a specific outgroup can generalise and improve attitudes toward the members of a broader range of social groups^43^. Our predictions are hence based on the idea that experiential contact itself is an important psychological factor in shaping attitudes. We also assume that contact with pets is likely to play a role in this process as pet contact is common and embedded in the lives of pet owners.

Second, we explore if identification with animals (and which of its three dimensions; solidarity with animals, human-animal similarity, animal pride) act as mechanisms through which positive contact with pets may lead to more positive and inclusive concerns for animals, nature, and human outgroups (Fig. 1). Given the lack of prior research specifically investigating the role of identification with animals as a mediator between contact with pets and the outcomes assessed herein, we adopted an exploratory perspective in terms of its mediating role. We did, however, make some predictions regarding which forms (i.e., dimensions) of identification with animals^16,39^ would be especially related to our different dependent variables. With respect to solidarity with animals, given that this dimension of identification is defined by the feeling of being connected to other animals and the desire to help and care for them, we expected it would be associated with more positive attitudes toward non-pet animals, along with higher environmental concerns (particularly those impacting animals) and a lower endorsement of meat consumption (i.e., lower usual meat consumption and a lesser endorsement of the beliefs that legitimize the consumption of meat). Given that solidarity with animals has also been associated with a more inclusive and egalitarian mindset^16,39^, we expected that this dimension of identification would be associated with more positive intergroup attitudes (i.e., weaker ingroup bias involving social groups and a lower endorsement of the social dominance orientation).

With respect to human-animal similarity, a dimension of identification which involves perceiving that animals share similarities and valued characteristics with humans, we expected it to be associated with more positive attitudes toward non-pet animals, along with a higher recognition of the interdependence that exists between humans, animals, and the environment. As per animal pride, because this dimension involves explicitly recognizing and valuing being an animal oneself, and that it has been associated with endorsing more competitive and hierarchical intergroup orientations (i.e., higher social dominance orientation and nationalism^16^), we expected that it would also be associated with higher ingroup bias involving human social groups and a higher endorsement of the social dominance orientation, along with a stronger endorsement of meat consumption, which can be seen as a ‘typical’ animal behavior (i.e., higher usual meat consumption and stronger endorsement of beliefs that legitimize meat consumption).

To maximise their generalizability, these associations were examined in a large representative dataset. Furthermore, a first poststratification weight variable was used to adjust the current sample to the general Canadian population on relevant sociodemographic variables. Because a growing number of studies are finding that pet owners are not equivalent to non-pet owners in terms of sociodemographic characteristics^44,45^, a second poststratification weight variable, which specifically adjusts for each subgroup’s (i.e., pet vs. non-pet owners) representativeness relative to the overall Canadian population, was used to minimise the impact of these potential differences. Given the topic of the study, quotas were also used to ensure that the proportions of pet and non-pet owners recruited for the study align with those observed in the Canadian population^1^.

## Results

### Participants and sociodemographic factors

Data from a total of 1069 participants were analyzed, which included 52.6% (562/1069) males and 47.0% (502/1069) females (5 indicated ‘other’ for their gender). Whereas 868 participants (81.2%) completed the questionnaire in English, 201 (18.8%) completed it in French. Approximately 46% of the sample were older than 50 years. More details on the sociodemographic characteristics of the sample are presented in Supplementary Table S1.

As can be seen in Supplementary Table S1, compared to non-pet owners, a significantly higher proportion of pet owners were found in the 30–34 age group, but lower proportions were found in the 65–64 and 70–74 age groups. In terms of living arrangements and area, and compared to non-pet owners, higher proportions of pet owners lived in a house or in the countryside, and lower proportions lived in an apartment/condo or in the city. In terms of gross annual household income, a significantly higher proportion of pet owners were found in the $160,000–$179,999 and $180,000–$199,999 ranges, but a lower proportion was found in the less than $20,000 range. In terms of employment, a higher proportion of pet owners compared to non-pet owners worked full-time, but a lower proportion were retired. As per ethnicity, a higher proportion of pet owners compared to non-pet owners were White/Caucasian, but lower proportions were of South Asian or Chinese ethnicities. In terms of marital status, and compared to non-pet owners, a higher proportion of pet owners were in a common-law union or selected ‘other’, but a lower proportion was single. Pet owners had a higher number of children currently living at home than non-pet owners. Many of the differences observed between pet and non-pet owners on these sociodemographic variables align with prior research^44–49^. When conducting the X^2^ and t-test analyses by applying the second postratification weight, the differences observed between pet and non-pet owners became non-significant, confirming that applying this poststratification weight ensures that comparisons of pet vs. non-pet owners are not confounded by sociodemographic differences.

### Comparison of pet and non-pet owners on the main variables

As seen in Table 1, pet owners reported higher identification with animals on all three dimensions, namely human-animal similarity, solidarity with animals, and animal pride, compared to non-pet owners. Pet owners also reported higher positive contact with pets and more positive attitudes toward non-pet animals, compared to non-pet owners. In terms of the intergroup relations outcomes, pet owners had more positive attitudes towards outgroups than non-pet owners. When comparing pet and non-pet owners on the environmental attitudes outcomes, pet owners reported higher biospheric environmental concerns than non-pet owners and stronger beliefs in human–environment interdependence. Finally, in terms of meat consumption, pet owners reported consuming fewer portions of meat weekly on average than non-pet owners.

**Table 1:** Results of independent sample T-tests comparing pet and non-pet owners on the dimensions of identification, positive contact with pets, positive attitudes toward non-pet animals, and the intergroup relations, environmental attitudes, and meat consumption outcomes.

	Pet Owners		Non-Pet Owners		Pet Ownership Effect			
	M	SD	M	SD	df	t	Cohen’s d	*p*
Human-animal similarity	4.10	1.34	3.71	1.28	1067	4.78	0.30	< .001
Solidarity with animals	5.01	1.21	4.00	1.25	1067	13.28	0.82	< .001
Animal pride	4.17	1.57	3.56	1.51	1067	6.41	0.40	< .001
Positive contact with pets	5.07	0.96	2.90	1.01	1067	35.77	2.22	< .001
Positive attitudes toward non-pet animals	4.66	0.99	4.09	0.90	1017	9.81	0.60	< .001
**Intergroup relations**								
Positive attitudes toward outgroups	63.48	20.67	60.76	19.87	1067	2.16	0.13	.031
Positive attitudes toward ingroups	79.18	19.53	76.86	19.28	1067	1.93	0.12	.054
Social dominance orientation	2.86	1.10	2.92	1.06	1067	−0.92	0.06	.356
**Environmental attitudes**								
Biospheric environmental concerns	5.85	0.97	5.47	1.24	821	5.38	0.35	< .001
Egoistic environmental concerns	5.54	1.11	5.54	1.16	1065	0.05	0.00	.961
Altruistic environmental concerns	5.86	1.13	5.83	1.18	1067	0.46	0.03	.644
Inclusion of nature in the self	4.42	1.66	4.31	1.67	1040	0.98	0.06	.327
Human–environment interdependence beliefs	4.29	0.72	4.12	0.79	913	3.64	0.23	< .001
**Meat consumption**								
Number of weekly meat portions	5.40	3.91	6.15	5.00	821	−2.65	0.17	.008
Diet	5.21	1.23	5.27	1.11	1067	−0.74	0.05	.457
Rationalization of meat consumption	4.41	1.23	4.53	1.16	1067	−1.65	0.10	.100

The second poststratification weight was applied in these analyses.

### Correlations among the main variables

As seen in Table 2, positive contact with pets showed significant correlations with the outcome variables. Specifically, positive contact with pets was associated with more positive attitudes toward non-pet animals, and more positive attitudes toward both ingroups and outgroups. Positive contact with pets was also negatively correlated with the social dominance orientation. For the environmental attitudes variables, positive contact with pets correlated with higher biospheric environmental concerns, inclusion of nature in the self, and human–environment interdependence beliefs. This pattern of correlations suggests that positive contact with pets is associated with most of the same outcomes, and in the same directions, as pet ownership.

**Table 2:** Correlations between the dimensions of identification, positive contact with pets, positive attitudes toward non-pet animals, and the intergroup relations, environmental attitudes, and meat consumption outcomes.

		*M*	*SD*	1	2	3	4	5	6	7	8	9	10	11	12	13	14	15
1	Human-animal similarity	3.92	1.31	–														
2	Solidarity with animals	4.56	1.31	.61***	–													
3	Animal pride	3.90	1.57	.72***	.64***	–												
4	Positive contact with pets	4.07	1.47	.30***	.56***	.34***	–											
5	Positive attitudes toward non-pet animals	4.40	1.00	.30***	.49***	.31***	.41***	–										
6	Positive attitudes toward outgroups	63.08	20.25	.14***	.15***	.14***	.13***	.22***	–									
7	Positive attitudes toward ingroups	79.26	18.70	.11***	.13***	.11***	.17***	.20***	.68***	–								
8	Social dominance orientation	2.88	1.10	−.13***	−.15***	−.08*	−.10**	−.18***	−.38***	−.29***	–							
9	Biospheric environmental concerns	5.68	1.07	.23***	.42***	.27***	.28***	.42***	.34***	.30***	−.37***	–						
10	Egoistic environmental concerns	5.57	1.08	.05	.09**	.03	−.00	−.03	.20***	.33***	−.07*	.41***	–					
11	Altruistic environmental concerns	5.90	1.11	.09**	.14***	.08*	.06	.11***	.37***	.48***	−.29***	.58***	.68***	–				
12	Inclusion of nature in the self	4.39	1.66	.13***	.18***	.11***	.15***	.16***	.12***	.13***	−.11***	.20***	.10**	.10**	–			
13	Human–environment interdependence beliefs	4.22	0.76	.18***	.29***	.19***	.20***	.24***	.27***	.33***	−.44***	.62***	.28***	.49***	.20***	–		
14	Number of weekly meat portions	5.88	4.83	.03	−.05	.04	−.04	−.12***	−.07*	−.04	.11***	−.19***	−.05	−.15***	.03	−.17***	–	
15	Diet	5.29	1.16	−.05	−.12***	−.07*	−.01	−.11***	−.04	.08*	.09**	−.14***	.01	−.02	−.06*	−.06*	.35***	–
16	Rationalization of meat consumption	4.50	1.21	−.11***	−.09**	−.12***	−.05	−.19***	−.14***	−.04	.32***	−.20***	.05	−.09**	−.09**	−.18***	.32***	.52***

*p < 0.05; **p < 0.01; ***p < 0.001. The first poststratification weight was applied in these analyses.

### Multiple regressions predicting the outcome variables

Multiple regressions were conducted to test whether the dimensions of identification with animals predict the outcome variables (Table 3). All tolerance values were above 0.42 and all variance inflation factors values (VIF) were equal to or less than 2.40, indicating that multicollinearity was not a problem in these analyses. Overall, solidarity with animals was the most consistent predictor of the outcome variables. Higher solidarity with animals independently predicted more positive contact with pets, more positive attitudes toward non-pet animals, more positive attitudes toward outgroups and ingroups, a lower social dominance orientation, as well as higher biospheric, egoistic, and altruistic environmental concerns, higher inclusion of nature in the self, and higher beliefs in human–environment interdependence. Higher solidarity also predicted the adoption of diets that include less meat and a smaller number of weekly meat portions consumed on average. Higher human-animal similarity independently predicted a lower social dominance orientation. Higher animal pride independently predicted a higher number of weekly meat portions consumed on average.

**Table 3:** Multiple regressions predicting positive attitudes toward non-pet animals, and the intergroup relations, environmental attitudes, and meat consumption outcomes.

	Dimensions of identification			
	Human-animal similarity	Solidarity with animals	Animal pride	
	β [95% CI]	β [95% CI]	β [95% CI]	*R*^*2*^
Positive attitudes toward non-pet animals	.00 [−.057, .063]	.50*** [.323, .431]	−.01 [−.057, .046]	.24***
**Intergroup relations**				
Positive attitudes toward outgroups	.05 [−.548, 2.214]	.09* [.181, 2.650]	.05 [−.608, 1.756]	.03***
Positive attitudes toward ingroups	.04 [−.714, 1.846]	.09* [.169, 2.457]	.03 [−.794, 1.398]	.02***
Social dominance orientation	−.12** [−.175, −.024]	−.13*** [−.177, .−042]	.09 [−.001, .128]	.03***
**Environmental attitudes**				
Biospheric environmental concerns	−.05 [−.106, .029]	.43*** [.290, .411]	.03 [−.039, .076]	.18***
Egoistic environmental concerns	.02 [−.056, .094]	.11** [.027, .160]	−.06 [−.103, .024]	.01*
Altruistic environmental concerns	.03 [−.053, .098]	.15*** [.055, .190]	−.03 [−.088, .041]	.02***
Inclusion of nature in the self	.04 [−.059, .170]	.17*** [.106, .309]	−.03 [−.125, 071]	.03***
Human–environment interdependence beliefs	.01 [−.044, .056]	.28*** [.116, .206]	.00 [−.041, .045]	.08***
**Meat consumption**				
Number of weekly meat portions	.05 [−.163, .501]	−.14*** [−.812, −.219]	.09* [.006, .574]	.01**
Diet	.05 [−.033, .125]	−.13** [−.187, −.045]	−.03 [−.088, .047]	.02***
Rationalization of meat consumption	−.05 [−.126, .040]	−.01 [− .079, .069]	−.08 [−.134, .008]	.02**

*p < 0.05; **p < 0.01; ***p < 0.001. The first poststratification weight was applied in these analyses.

### Mediation analyses testing the underlying mechanisms

To test the possible mechanisms through which positive contact with pets impacts people’s attitudes toward non-pet animals, intergroup relations involving human groups, attitudes toward the environment, and meat consumption, mediation analyses were conducted. Specifically, and as per Fig. 1, the three dimensions of identification with animals (i.e., solidarity with animals, human-animal similarity, animal pride) were tested as mediators in the association between positive contact with pets and the outcome variables. Positive contact with pets was included as the independent variable in these analyses given that this specific indicator of contact provides a more inclusive test of the pets as ambassadors effect, that its continuous scale presents a better fit with the mediation analyses, and that this indicator shows the same general pattern of relationships relative to the pet ownership variable. A bootstrapping procedure with a 95% confidence interval (CI) and 5,000 bootstraps samples was used in these analyses. The direct effects observed in the mediation analyses are reported in Table 4; the total, total indirect, and indirect effects are reported in Supplementary Tables S2-S5. The significant indirect effects observed are reported below.

**Table 4:** Direct effects observed in the mediation models predicting the outcome variables.

	Predictors											
	Positive contact with pets			Human-Animal Similarity			Solidarity with Animals			Animal Pride		
	β [95% CI]	SE	*p*	β [95% CI]	SE	*p*	β [95% CI]	SE	*p*	β [95% CI]	SE	*p*
Human–Animal Similarity	**.30 [.219, .381]**	**.04**	**.000**									
Solidarity with Animals	**.56 [.499, .623]**	**.03**	**.000**									
Animal Pride	**.34 [.258, .407]**	**.04**	**.000**									
Positive attitudes toward non-pet animals	**.20 [.110, .287]**	**.05**	**.000**	.02 [−.082, .115]	.05	.762	**.38 [.256, .483]**	**.06**	**.000**	−.01 [−.105, .092]	.05	.877
**Intergroup relations**												
Positive attitudes toward outgroups	.07 [− .036, .157]	.05	.186	.06 [−.061, .177]	.06	.341	.05 [−.075, .177]	.06	.412	.05 [−.084, .176]	.07	.505
Positive attitudes toward ingroups	**.14 [.044, .235]**	**.05**	**.005**	.05 [−.072, .171]	.06	.440	.01 [−.118, .131]	.06	.891	.03 [−.093, .154]	.06	.679
Social dominance orientation	−.03 [−.124, .076]	.05	.599	−.**12 [**−.**244, **−.**001]**	**.06**	**.055**	−.12 [−.239, .017]	.07	.079	.09 [−.027, .201]	.06	.121
**Environmental attitudes**												
Biospheric environmental concerns	.06 [−.036, .154]	.05	.216	−.04 [−.140, .053]	.05	.377	**.39 [.277, .504]**	**.06**	**.000**	.03 [−.081, .132]	.05	.608
Egoistic environmental concerns	−.08 [−.173, .022]	.05	.121	.02 [−.101, .131]	.06	.757	**.16 [.033, .277]**	**.06**	**.010**	−.06 [−.169, .059]	.06	.317
Altruistic environmental concerns	−.03 [−.127, .059]	.05	.486	.03 [−.081, .137]	.06	.658	**.17 [.056, .274]**	**.06**	**.003**	−.03 [−.139, .073]	.06	.536
Inclusion of nature in the self	.08 [−.024, .187]	.05	.136	.05 [−.064, .161]	.06	.407	.12 [−.020, .252]	.07	.093	−.03 [−.144, .098]	.06	.680
Human–environment interdependence beliefs	.06 [−.034, .147]	.05	.205	.01 [−.091, .117]	.05	.792	**.24 [.127, .356]**	**.06**	**.000**	.01 [−.103, .114]	.06	.931
**Meat consumption**												
Number of weekly meat portions	−.01 [−.100, .089]	.05	.865	.05 [−.079, .159]	.06	.461	−.**14 [**−.**246, **−.**031]**	**.06**	**.014**	.09 [−.019, .212]	.06	.107
Diet	.08 [−.037, .185]	.06	.158	.06 [−.058, .177]	.06	.347	−.**18 [**−.**326, **−.**030]**	**.08**	**.018**	−.03 [−.164, .103]	.07	.688
Rationalization of meat consumption	−.01 [−.117, .101]	.06	.859	−.05 [−.168, .079]	.06	.460	.00 [−.146, .141]	.07	.993	−.08 [−.203, .044]	.06	.197

The first poststratification weight was applied in these analyses.

Significant values are in [bold].

As seen in Table 4, contact with pets again significantly and positively predicted each of the three dimensions of identification in these analyses. Tests of the indirect effects first revealed a significant effect of positive contact with pets on positive attitudes toward non-pet animals, via solidarity with animals (*β* = 0.21, SE = 0.04, 95% CI = [0.137, 0.287]): more positive contact with pets was associated with higher solidarity with animals which, in turn, was associated with more positive attitudes toward non-pet animals. In terms of the *intergroup relations* variables, tests of the indirect effects revealed a significant effect of positive contact with pets on social dominance orientation, via human-animal similarity (*β* = − 0.04, SE = 0.02, 95% CI = [− 0.077, − 0.001]): more positive contact with pets was associated with higher human-animal similarity which, in turn, was associated with lower social dominance orientation.

As per the *environmental attitudes* outcomes, tests of the indirect effects revealed a significant effect of positive contact with pets on the biospheric, egoistic, and altruistic environmental concerns, via solidarity with animals (respectively, *β* = 0.22, SE = 0.04, 95% CI = [0.151, 0.299]; *β* = 0.09, SE = 0.04, 95% CI = [0.019, 0.161]; *β* = 0.09, SE = 0.03, 95% CI = [0.033, 0.160]): more positive contact with pets was associated with higher solidarity with animals which, in turn, was associated with higher biospheric, egoistic, and altruistic environmental concerns. Moreover, tests of the indirect effects revealed a significant effect of positive contact with pets on human–environment interdependence beliefs, via solidarity with animals (*β* = 0.14, SE = 0.03, 95% CI = [0.072, 0.206]): more positive contact with pets was associated with higher solidarity with animals which, in turn, was associated with stronger beliefs human–environment interdependence.

In terms of the *meat consumption* outcomes, tests of the indirect effects revealed a significant effect of positive contact with pets on number of weekly meat portions consumed, via solidarity with animals (*β* = − 0.08, SE = 0.03, 95% CI = [− 0.142, − 0.019]): more positive contact with pets was associated with higher solidarity with animals which, in turn, was associated with fewer meat portions consumed weekly. Moreover, tests of the indirect effects revealed a significant effect of positive contact with pets on diet, via solidarity with animals (*β* = − 0.10, SE = 0.04, 95% CI = [− 0.189, − 0.017]): more positive contact with pets was associated with higher solidarity with animals which, in turn, was associated with diets containing less meat.

## Discussion

While a majority of people in different occidental countries live with a least one pet, whether our contact with pets have implications for non-pet animals, other living entities, and social groups that go beyond the specific context of human-pet relations needs further investigation. To this aim, the current study tested an extended pets as ambassador hypothesis, and whether humans’ proximal and concrete contacts with pets have implications for attitudes toward a diversity of non-pet animals (i.e., farm, wild, meat-animals), nature, and fellow humans.

Two indicators of contact with pets were assessed, namely: pet ownership (a more objective indicator) and frequency of positive contact with pets (a more subjective indicator, not restricted to current pet owners). The current study also explored the psychological processes through which these broadening effects take place. Specifically, we tested if social identification with animals—a particularly inclusive type of social identity—could act as a mechanism through which contacts with pets exerts its beneficial effect beyond the specific context of human-pet relations. These associations were tested in a large representative sample. To maximise the generalisability of the findings, account for potentially confounding sociodemographic factors, and maximize the comparability of the pet and non-pet owner subgroups, all statistical analyses included a poststratification weight.

When comparing pet and non-pet owners on the outcome variables assessed, we found that pet owners reported more positive attitudes toward non-pet animals and more positive contact with pets. Pet owners also reported more positive attitudes toward human outgroups, higher biospheric environmental concerns (i.e., for plants and animals), higher beliefs in human–environment interdependence, and a lower number of meat portions eaten weekly. These findings suggest that pet ownership may contribute to broadening people’s concerns for a diversity of social groups and living entities. In line with prior work^16^, pet owners also identified with animals to a great extent on the three dimensions of identification, compared to non-pet owners. Correlations observed between the frequency of positive contact with pets and the outcomes revealed a similar picture. More frequent positive contacts with pets was associated with: more positive attitudes toward non-pet animals, more positive attitudes both toward ingroups and outgroups, a lower endorsement of social dominance orientation, and higher biospheric environmental concerns, greater beliefs in human–environment interdependence, as well as a higher inclusion of nature in the self.

To capture the psychological processes that may account for the associations between the positive contact with pets variable and the outcomes, we next examined which dimensions of identification with animals predict these outcomes. Overall, solidarity with animals emerged as the most consistent predictor of the outcome variables, predicting: more positive attitudes toward non-pet animals, more positive attitudes toward human outgroups and ingroups but a lower social dominance orientation, as well as higher environmental concerns and inclusion of nature in the self, greater beliefs in human–environment interdependence, and lower meat consumption. Human-animal similarity specifically predicted a lower social dominance orientation. Animal pride predicted a higher number of weekly meat portions eaten. These findings align with prior research, generally showing a particularly potent role for solidarity with animals in predicting pro-social outcomes for animals and the endorsement of a more equalitarian outlook on intergroup relations involving human social groups^16,39^. Yet, the current findings go beyond this prior work by extending these beneficial outcomes to the realm of the environment and by targeting particularly diverse social groups.

Building on these findings, mediation analyses then tested which dimension of identification with animals acts as a process through which positive contact with pets exerts its beneficial effects observed on some of the outcome variables. Solidarity also emerged as the clearer mediator in these analyses, as it significantly mediated the associations between positive contact with pets and: positive attitudes toward non-pet animals; biospheric, egoistic, and altruistic environmental concerns; human–environment interdependence beliefs; diet; and number of weekly meat portions consumed. The similarity dimension also emerged as a significant mediator in the association between frequent positive contact with pets and social dominance orientation. Given that this dimension involves perceiving greater similarity between humans and animals and bringing animals (i.e., a typically less valued group) closer to humans (i.e., perceived as a higher status group;^16^), individuals high in human-animal similarity might also perceive greater similarity between human groups per se; as a result, these individuals may hence be less likely to support a hierarchy that differentiates between human social groups but more motivated to ‘equalise’ the status of lower and higher status groups.

Taken together, these findings contribute to prior research on the pets as ambassador effect by investigating a wide and extended range of outcomes (i.e., involving the natural environment and meat consumption, and human social groups), as well as further testing the psychological mediators in the association between contact with pets and these outcomes. The results suggest that our contacts with pets, as a particularly proximal, concrete, and prototypical type of human-animal relation, could serve as a basis for developing broader social concerns. Importantly, and providing a strong test for the pets as ambassador effect, we found that the frequency of positive contact with pets—i.e., as a more subjective indicator of contact with pets, not restricted to current pet owners—played a significant role in broadening people’s concerns for the environment, animals, and human social groups. This suggests that owning a pet per se, or being naturally inclined to gravitate toward pets, may not be required to promote these broader concerns; simply having positive and frequent contacts with pets may be enough. This observation is important in light of the demands associated with pet ownership^46^ and the stress that may come with caring for pets^50,51^. Furthermore, the carbon footprint associated with owning pets has recently been estimated^52^. While the findings uncovered herein suggest benefits to our contacts with pets in terms of promoting higher environmental concerns, whether the benefits of these contacts are sufficient to offset the objective environmental consequences of owning and caring for pets should be tested systematically in future research.

Because of the methodological and statistical procedures employed, our findings can be generalized to the Canadian population. The comparison of pet and non-pet owners was also rigorous due to our approach of statistically adjusting each of these two subgroups (i.e., pet and non-pet owners) to the overall Canadian population. While the effect sizes for some of our statistically significant associations and effects are considered of small magnitude (e.g., correlations of |.10|; Cohen’s d of 0.20 or below), they do point to what appears to be a consistent and generalizable trend. Nevertheless, and although different strategies were employed during the recruitment to maximise and facilitate participation, the fact that the current study was conducted online may have limited access to some segments of the Canadian population.

Given the cross-sectional nature of the current study and that the current findings were associative and not causal, future research should employ longitudinal and/or experimental designs (using ethically sound procedures) to further test the direction of causality between contact with pets variable and the outcomes. For example, longitudinal studies conducted prior to and during the process of adopting a new pet could directly test if pre-existing positive attitudes toward animals in general and toward the environment predict an increased likelihood of adopting a new pet, or if adopting a new pet predicts an increase in positive attitudes toward animals in general and toward the environment. Furthermore, future cross-cultural research would be needed to test the generalizability of the current findings beyond the specific Canadian context, and the possible boundary conditions of the observed effects. Attitudes towards pets and the functions fulfilled by pets vary widely across cultures^53,54^. These cultural variations in human-pet relationships could potentially influence the capacity of pets to serve as ambassadors for other animal species and human groups. Indeed, it is possible that a cultural representation of pets as capable of promoting acceptance and inclusion, as well as of widening social concerns, may have been shared among the current participants and could account for some of the associations observed herein. As well, in countries where resources shared among social groups are particularly scarce, contact with pets (and other animals) may be perceived as further compromising access to these resources rather than promoting heightened social concerns^53^.

In sum, this research provided a systematic test for an extended pets as ambassador effect, whereby our contacts with pets have implications for a diversity of (non-pet) animal types, nature, and for human social groups. The present findings provide an investigation of the broader benefits associated with the presence of pets in our human lives, and how our contacts with pets—as a particularly accessible and concrete type of human-animal relation—can serve as a springboard toward broader social concerns. We hope that the data-driven approach adopted herein, along with the rigorous methodological and statistical approaches employed, will continue raising research interest in human-pet relationships.

## Methods

### Recruitment

Results were based on a nationally representative survey of 1,069 Canadian adults (18 and older), conducted by the survey firm Léger from February 1 to February 12, 2022. Based on Canada’s total population (38 million), this sample size involves a margin of error of 3% and a 95% confidence level. Given the topic of the study, quotas were imposed to recruit 619 pet owners and 449 non-pet owners and ensure that the percentage of pet owners in the sample aligns with the percentage observed in the Canadian population^1^; the final sample included 619 pet owners and 450 non-pet owners. Participants were invited to participate in the study via an email sent by Léger. All invitations were bilingual and participants could complete the questionnaire in either French or English, which are the two official languages in Canada. Respondents were drawn from Léger’s LEO internet panel, a widely used national probability-based online panel that includes over 420,000 active members in Canada. Within this panel, 65% of the profiles have been updated in the last six months, and 50% of the profiles are based on the Statistics Canada census. Most of Léger's panel members have been randomly recruited by telephone over the past decade, making the panel highly representative of the Canadian population; another third of the panelists were recruited by third parties or through various partnership and advertising programs. The remainder chose to register via Léger’s social media platforms (Facebook and Twitter). Léger panels have been used in other peer-reviewed academic research^55–58^.

Léger administered the Qualtrics-based online questionnaire. The following measures were taken to maximize participation and the representativity of the final sample: (1) the survey was open for two weeks, so as to leave sufficient time for participants to open the invitation email and complete the questionnaire; (2) the email invites were sent in waves over this 2-week data collection period; (3) participants could pause the survey and continue later, starting exactly where they left off before taking a break, without losing their data; (4) the survey was accessible 24 h a day, seven days a week, from any Internet-connected computer and handheld device (tablets and smartphones). Using a cross-platform approach ensures that highly mobile people, more often younger people, participate in greater numbers^59,60^; (5) in the event of any technical problems, respondents could email the technical support team or contact Leger directly by phone. Léger’s technical support team was available throughout the fieldwork to help address difficulties, if any; (6) reminder emails were sent to all individuals who were invited to participate but had not yet taken the survey.

A total of 8,501 email invitations were sent to panel members, of whom 1326 opened the invitation email. Among those, 32 refused to take part in the study, and 10 participants were considered non-eligible (i.e., 3 were non-eligible on the basis of their age; 7 failed one of the two attention check questions), 69 exceeded the quotas fixed, and 146 had incomplete data (i.e., they did not reach the end of the questionnaire). This resulted in 1069 qualified completes used for analysis. When considering the total number of email invitations sent to potential participants, the participation rate is 13%; when not considering the individuals who have not opened the invitation email in this calculation, the participation rate is 81%. Median response time among qualified completes was 19 min. Participants were paid the equivalent of CAN$3 directly by Léger for participating in this study. Participants’ compensation takes the form of points that can be redeemed from different merchants (e.g., Starbucks, Tim Hortons) and entry into a draw (full details available via: www.legeropinion.com/fr/recompenses/). Informed consent was obtained from all participants. The study was approved by the Ethics Committee involving Human Participants of the University of Québec in Montréal (certificate number 2012–372). We confirm that the research and all methods were performed in accordance with the guidelines of the Canadian Tri-Council Policy for the Ethical Conduct of Research Involving Humans.

### Poststratification weights

Two poststratification statistical weights were prepared by Léger and provided to the research team, and then used in the main statistical analyses to account for differences between our sample and the 2016 Canadian Census benchmarks. Based on data from Statistics Canada, the following benchmark distributions of Canadians who are 18 years and older from the general population were used to compute the two poststratification weights: gender, age, Province of residence, area lived in (rural, urban, countryside), mother tongue, education, presence of children in the household, marital status, type of dwelling, ethnicity, gross annual household income, employment status. These sociodemographic variables were chosen on the basis of their utility for adjusting the current sample to the general Canadian population, and of recommendations for conducting research comparing pet and non-pet owners^44,61^. Furthermore, the following sociodemographic factors have been associated with attitudes toward animals and animal-related behaviors: sex/gender^35,62–66^; age^35,62,65,67^; education^65,68–70^; area lived in^65^; income^71,72^.

The first poststratification statistical weight was used to adjust the current sample to the general Canadian population on these sociodemographic variables. This first poststratification weight variable was included in the analyses conducted on the entire sample. The second poststratification weight variable adjusts both the pet and non-pet owners subgroups to the general Canadian population on these same sociodemographic variables; doing so ensures that both subgroups are each adjusted idiosyncratically to the Canadian population and maximises these subgroups’ comparability. This second poststratification weight was included in the analyses that involved comparing pet and non-pet owners. Together, these methodological and statistical procedures allow for the improved generalization of findings, and ensure that comparisons of pet vs. non-pet owners are not confounded by sociodemographic differences.

### Questionnaire and measuring instruments

The measuring instruments were translated from English to French using a back-to-back translation procedure or a committee approach. When conducting this translation, the research assistants were instructed by the lead researcher to give priority to loyalty of meaning and familiarity of the content instead of strict loyalty to the original language (i.e., a decentering approach^73^). All the measures used for this study have been selected on the basis of their validity and established psychometric properties. The measures reported for the current study were taken from a larger representative survey and dataset pertaining to perceptions of animals and humans, identification with animals, social attitudes, and current issues.

#### Sociodemographic and pet ownership information

The sociodemographic data included the following categories: language of questionnaire (English or French), gender (male, female, other), age (18–21, 22–24, 25–29, 30–34, 35–39, 40–44, 45–49, 50–54, 55–59, 60–64, 65–69, 70–74,  > 75 years), education level (Primary school diploma, High school diploma, Diploma of Collegial studies (e.g., CEGEP), Certificates and Diplomas (University), Bachelor's degree, Master's degree, Doctoral degree, Other), dwelling type (apartment/condo, house, other), area lived in (city, suburb, countryside), gross annual household income (less than $20,000, $20,000–$39,999, $40,000–$59,999, $60,000–$79,999, $80,000–$99,999, $100 000–$119,999, $120,000–$139,999, $140,000–$159,999, $160,000–$179,999, $180,000–$199,999, over $200,000), employment (full-time, part-time, self-employed, student, homemaker, unemployed, retired), ethnicity (White, South Asian, Chinese, Black, Filipino, Arab, Latin American, Southeast Asian, West Asian, Korean, Japanese, Other), marital status (common-law union, married, separated, divorced, single, widowed, single parent, Other), number of children currently living at home, political orientation on social and economic issues (on a scale from 1 (*Left/Liberal*) to 7 (*Right/Conservative*)). To measure pet ownership, participants were asked: “Do you have one or more pet(s) currently?” (yes, no).

#### Attitudes toward and relations with animals

*Social identification with animals* was assessed using the Identification with Animal Measure (IWAM^16^). The 15-item measure is designed to assess how humans identify with animals as a group on three dimensions: human-animal similarity (e.g., ‘Animals, including human animals, are very similar to each other’; α = 0.86), solidarity with animals (e.g., ‘I feel solidarity with animals’; α = 0.90), and animal pride (e.g., ‘I am proud to be an animal’; α = 0.96). Responses were made on a 1 (*not agree at all*) to 7 (*very strongly agree*) scale.

*Positive contact with pets* was assessed using three items from Auger and Amiot^20,41^ (e.g., ‘You spend time with pets’; α = 0.96), and two additional items assessing time spent with pets and activities done with pets; this measure hence covers both quantitative (i.e., frequency) and qualitative (i.e., reciprocity and affective valence) aspects of contact with pets (see^74–76^). Items were rated on a scale from 1 (*never*) to 6 (*always*).

*Positive attitudes toward non-pet animals* were measured by asking participants to indicate to what extent they like or dislike animals from 12 different species; these species were selected given their relevance to the Canadian population^20^. Based on the instructions used in prior large-scale studies^77,78^, participants were asked to indicate their liking of each animal species on a scale from 1 (*strongly dislike*) to 7 (*strongly like*). These 12 animal species were then regrouped into the variable representing positive attitudes toward non-pet animals (i.e., composite score including wild animals, pests, and farm animals; α = 0.90).

#### Intergroup relations

To measure *positive attitudes toward outgroups* and *positive attitudes toward ingroup*s, participants were presented with a list of different social groups; four represented outgroups (e.g., ‘People with different religious beliefs’; α = 0.84) and two represented ingroups (e.g., ‘Members of your family’; α = 0.67). Participants were asked to refer to a feeling thermometer ranging from 1 (*negative or cold feelings*) to 100 (*positive feelings*), and to rate their attitudes toward each group using a slider.

*Social dominance orientation* was assessed using the SDO_7(s)_ scale^79^. The scale is designed to measure participants’ preferences for group-based hierarchy (e.g., ‘Group equality should not be our primary goal’; α = 0.83). Responses were made on a 1 (*strongly oppose*) to 7 (*strongly favor*) scale.

#### Environmental attitudes

Environmental concerns were evaluated with 15 items from Schultz^30^. This measure captures participants’ concerns toward different living entities as a result of environmental problems: *biospheric environmental concerns* (e.g., ‘Marine life’, ‘Plants’; α = 0.94), *egoistic environmental concerns* (e.g., ‘Me’, ‘My future’; α = 0.89), and *altruistic environmental concerns* (e.g., ‘Humanity’, ‘Future generations’; α = 0.88). Participants rated each item on a scale from 1 (*not important*) to 7 (*supreme importance*). The *Inclusion of Nature in the Self* (INS) scale assessed participants’ interconnection with nature^80^. Participants 

were presented with a series of pictures representing two overlapping circles labelled ‘self’ and ‘nature’, and asked to circle the picture that best describes their relationship with the natural environment. Scores ranged from 1 (for circles that touched but did not overlap) to 7 (for circles that overlapped almost completely).

The five-item *New Human Interdependence Paradigm* (NHIP) scale was used to assess participants’ beliefs in human–environment interdependence (e.g., ‘Human beings can progress only by conserving nature’s resources’; α = 0.90^29^). Participants indicated their agreement with each item using a 1 (*strongly disagree*) to 5 (*strongly agree*) scale.

#### Meat consumption

To assess average *number of weekly meat portions*, participants were asked: ‘How many portions of meat do you eat per week (on average)?’. Participants were also asked to indicate which *diet* best described their eating habits using the following scale: vegan (1); vegetarian (2); pescetarian (3); semi-vegetarian/flexitarian (4); omnivore trying to reduce meat consumption (5); meat eater/omnivore (6).

*Rationalization of meat consumption* was assessed using the 16-item 4N Scale^38^, which measures the endorsement of four types of justifications that legitimize meat consumption: meat consumption being natural (e.g., ‘Our human ancestors ate meat all the time’), necessary (e.g. ‘A healthy diet requires at least some meat’), normal (e.g., ‘It is normal to eat meat’), and nice (e.g., ‘Meat is delicious’). Participants rated each item on a scale from 1 (*completely disagree*) to 7 (*completely agree*). Answers on the four subscales were combined to create a composite score, encompassing the four justifications for meat consumption (α = 0.91).

### Statistical analyses

The independent-samples t-tests, descriptive statistics, correlations, and multiple regressions were performed using SPSS (IBM SPSS Statistics for Windows, Version 28.0. Armonk, NY: IBM Corp.) The mediation analyses were conducted using the Mplus software (Version 8.8). Table 1 presents the results of the independent-samples t-tests conducted to compare pet and non-pet owners on the outcome measures; the data used for these analyses are weighted (using the second poststratification weight). Table 2 present the results of the correlational analyses; the data used for these analyses are also weighted (using the first poststratification weight). Table 3 presents the results from the multiple regressions; these analyses include the first poststratification weight. Table 4 and Supplementary Tables S2-S5 present the results from the mediation analyses; these analyses include the first poststratification weight.

### Open practices statement

This study was pre-registered on the Open Science Framework: https://osf.io/bqyp3/?view_only=25ae39dbd0e84df08769afc60c2baf26. The hypotheses pertaining to the associations between the three dimensions of identification with animals and the outcome variables were pre-registered, whereas the analyses involving the pet ownership and contact with pets variables were exploratory. The results of analyses conducted on additional dependent variables pertaining to the perceptions of animals and of humans that were assessed in this study are reported in the Supplementary Materials document (Supplementary Tables S6 to S10). The questionnaire measures used for the analyses reported in this paper are also available in the Supplementary Materials document.

### Supplementary Information

Supplementary Information.

## Data Availability

The datasets analysed during the current study are available in the Open Science Framework repository, https://osf.io/63v74/?view_only=19398a829f924880b527474fceb178bb. These datasets and codes support the findings presented in this manuscript and can be accessed for verification purposes only.
