# Supplementary material for: Exploring the role of our contacts with pets in broadening concerns for animals, nature, and fellow humans: a representative study
Source: Sci Rep. 2023 Oct 10;13:17079. doi: 10.1038/s41598-023-43680-z (PMC10564771; doi:10.1038/s41598-023-43680-z)
Supplement: Supplementary file 1 — Supplementary Information. [file 41598_2023_43680_MOESM1_ESM.docx]

Supplementary Information for:

**Exploring the Role of our Contact with Pets in Broadening Concerns for Animals, Nature, and Fellow Humans: A Representative Study**

Catherine E. Amiot^1^, Christophe Gagné^1^, and Brock Bastian^2^

^1^ Département de psychologie, Université du Québec à Montréal, C.P. 8888, Succ. Centre-Ville, Montréal, PQ, H3C 3P8, Canada

^2^ Melbourne School of Psychological Sciences, The University of Melbourne, 06, 05, Redmond Barry Building, Parkville, VIC 3010, Australia

* E-mail: amiot.catherine@uqam.ca, Tel.: (514) 987-3000, ext. 5006, Fax: (514) 987-7953

This supplementary information document includes Figure 1, Tables S1-S10, as well as the consent form and questionnaire measures employed in the current study. Table S1 reports the full sociodemographic information for the sample. Tables S2-S5 report the total, total indirect, and indirect effects for the mediation analyses presented in the article. Tables S6-S10 present the results of the analyses conducted on the positive contact with pets variable and on additional dependent variables pertaining to the perceptions of animals and of humans that were assessed in this study.

Identification with Animals:

Contact with Pets:

Outcomes:

- Pet Ownership
- Frequency of Positive Contact with Pets
- Solidarity with Animals
- Human-Animal Similarity
- Animal Pride
- Attitudes toward Non-Pet Animals
- Intergroup Relations
- Environmental Attitudes
- Meat Consumption

**Figure 1.** Mediation model tested, whereby the contact with pets variables (i.e., pet ownership and frequency of positive contact with pets) predict the dimensions of identification with animals (i.e., solidarity with animals, human-animal similarity, animal pride), which in turn predict the outcomes (i.e., attitudes toward non-pet animals, intergroup relations, environmental attitudes, meat consumption).

Table S1

*Sociodemographic Information for the Overall Sample and for Pet and Non-Pet Owners (Unweighted Data).*

|  |  | | Overall Sample | | Pet Owners (*N*=619) | | Non-Pet Owners (*N*=450) | |  |
| --- | --- | --- | --- | --- | --- | --- | --- | --- | --- |
|  |  | | *N* | % | *N* | % | *N* | % | Statistics |
| Language of questionnaire | English | | 868 | 81.2 | 498 | 80.5 | 370 | 82.2 | X^2^ (1) = 0.54  *p* = .465 |
|  | French | | 201 | 18.8 | 121 | 19.5 | 80 | 17.8 |  |
|  |  | | *N* | % | *N* | % | *N* | % |  |
| Gender | Male | | 562 | 52.6 | 309 | 49.9^a^ | 253 | 56.2^a^ | X^2^ (2) = 5.08  *p* = .079 |
|  | Female | | 502 | 47.0 | 308 | 49.8^a^ | 194 | 43.1^a^ |  |
|  | Other | | 5 | 0.5 | 2 | 0.3 | 3 | 0.7 |  |
|  |  | | *N* | % | *N* | % | *N* | % |  |
| Age | 18 to 21 years old | | 45 | 4.2 | 22 | 3.6 | 23 | 5.1 | X^2^ (12) = 35.98  *p* < .001 |
|  | 22 to 24 years old | | 65 | 6.1 | 37 | 6.0 | 28 | 6.2 |  |
|  | 25 to 29 years old | | 80 | 7.5 | 51 | 8.2 | 29 | 6.4 |  |
|  | 30 to 34 years old | | 104 | 9.7 | 75 | 12.1^a^ | 29 | 6.4^a^ |  |
|  | 35 to 39 years old | | 98 | 9.2 | 63 | 10.2 | 35 | 7.8 |  |
|  | 40 to 44 years old | | 85 | 8.0 | 53 | 8.6 | 32 | 7.1 |  |
|  | 45 to 49 years old | | 100 | 9.4 | 64 | 10.3 | 36 | 8.0 |  |
|  | 50 to 54 years old | | 124 | 11.6 | 76 | 12.3 | 48 | 10.7 |  |
|  | 55 to 59 years old | | 61 | 5.7 | 38 | 6.1 | 23 | 5.1 |  |
|  | 60 to 64 years old | | 95 | 8.9 | 46 | 7.4 | 49 | 10.9 |  |
|  | 65 to 69 years old | | 90 | 8.4 | 38 | 6.1^a^ | 52 | 11.6^a^ |  |
|  | 70 to 74 years old | | 70 | 6.5 | 32 | 5.2^a^ | 38 | 8.4^a^ |  |
|  | 75 or older | | 52 | 4.9 | 24 | 3.9 | 28 | 6.2 |  |
|  |  | | *N* | % | *N* | % | *N* | % |  |
| Education | Primary school diploma | | 18 | 1.7 | 9 | 1.5 | 9 | 2.0 | X^2^ (7) = 8.66  *p* = .278 |
|  | High school diploma | | 208 | 19.5 | 122 | 19.7 | 86 | 19.1 |  |
|  | Diploma of Collegial studies (e.g., CEGEP) | | 334 | 31.2 | 204 | 33.0 | 130 | 28.9 |  |
|  | University: Certificates and diplomas | | 76 | 7.1 | 45 | 7.3 | 31 | 6.9 |  |
|  | University: Bachelor's degree | | 294 | 27.5 | 167 | 27.0 | 127 | 28.2 |  |
|  | University: Master's degree | | 109 | 10.2 | 54 | 8.7 | 55 | 12.2 |  |
|  | University: Doctoral degree | | 19 | 1.8 | 9 | 1.5 | 10 | 2.2 |  |
|  | Other | | 11 | 1.0 | 9 | 1.5 | 2 | 0.4 |  |
|  |  | | *N* | *%* | *N* | *%* | *N* | *%* |  |
| Dwelling type | Apartment/Condo | | 310 | 29.0 | 138 | 22.3^a^ | 172 | 38.2^a^ | X^2^ (2) = 33.35  *p* < .001 |
|  | House | | 724 | 67.7 | 462 | 74.6^a^ | 262 | 58.2^a^ |  |
|  | Other | | 35 | 3.3 | 19 | 3.1 | 16 | 3.6 |  |
|  |  | | *N* | % | *N* | % | *N* | % |  |
| Area | City | | 506 | 47.3 | 267 | 43.1^a^ | 239 | 53.1^a^ | X^2^ (2) = 13.45  *p* = .001 |
|  | Suburb | | 369 | 34.5 | 221 | 35.7 | 148 | 32.9 |  |
|  | Countryside | | 194 | 18.1 | 131 | 21.2^a^ | 63 | 14.0^a^ |  |
|  |  | | *N* | % | *N* | % | *N* | % |  |
| Gross annual household income | Less than $20,000 | | 66 | 6.2 | 24 | 3.9^a^ | 42 | 9.3^a^ | X^2^ (10) = 26.14  *p* = .004 |
|  | $20,000 to $39,999 | | 141 | 13.2 | 79 | 12.8 | 62 | 13.8 |  |
|  | $40,000 to $59,999 | | 157 | 14.7 | 86 | 13.9 | 71 | 15.8 |  |
|  | $60,000 to $79,999 | | 145 | 13.6 | 91 | 14.7 | 54 | 12.0 |  |
|  | $80,000 to $99,999 | | 151 | 14.1 | 90 | 14.5 | 61 | 13.6 |  |
|  | $100,000 to $119,999 | | 101 | 9.4 | 63 | 10.2 | 38 | 8.4 |  |
|  | $120,000 to $139,999 | | 46 | 4.3 | 29 | 4.7 | 17 | 3.8 |  |
|  | $140,000 to $159,999 | | 70 | 6.5 | 41 | 6.6 | 29 | 6.4 |  |
|  | $160,000 to $179,999 | | 35 | 3.3 | 27 | 4.4^a^ | 8 | 1.8^a^ |  |
|  | $180,000 to $199,999 | | 26 | 2.4 | 20 | 3.2^a^ | 6 | 1.3^a^ |  |
|  | Over $200,000 | | 37 | 3.5 | 19 | 3.1 | 18 | 4.0 |  |
|  | Prefer not to answer | | 94 | 8.8 | 50 | 8.1 | 44 | 9.8 |  |
|  |  | | *N* | % | *N* | % | *N* | % |  |
| Employment | Full-time | | 502 | 47.0 | 325 | 52.5^a^ | 177 | 39.3^a^ | X^2^ (6) = 31.65  *p* < .001 |
|  | Part-time | | 93 | 8.7 | 53 | 8.6 | 40 | 8.9 |  |
|  | Self-employed | | 67 | 6.3 | 40 | 6.5 | 27 | 6.0 |  |
|  | Student | | 68 | 6.4 | 35 | 5.7 | 33 | 7.3 |  |
|  | Homemaker | | 43 | 4.0 | 30 | 4.8 | 13 | 2.9 |  |
|  | Unemployed | | 50 | 4.7 | 26 | 4.2 | 24 | 5.3 |  |
|  | Retired | | 246 | 23.0 | 110 | 17.8^a^ | 136 | 30.2^a^ |  |
|  |  | | *N* | % | *N* | % | *N* | % |  |
| Ethnicity | White | | 839 | 78.5 | 513 | 82.9^a^ | 326 | 72.4^a^ | X^2^ (11) = 30.37  *p* = .001 |
|  | South Asian | | 41 | 3.8 | 16 | 2.6^a^ | 25 | 5.6^a^ |  |
|  | Chinese | | 61 | 5.7 | 25 | 4.0^a^ | 36 | 8.0^a^ |  |
|  | Black | | 26 | 2.4 | 10 | 1.6 | 16 | 3.6 |  |
|  | Filipino | | 12 | 1.1 | 6 | 1.0 | 6 | 1.3 |  |
|  | Arab | | 11 | 1.0 | 4 | 0.6 | 7 | 1.6 |  |
|  | Latin American | | 13 | 1.2 | 6 | 1.0 | 7 | 1.6 |  |
|  | Southeast Asian | | 6 | 0.6 | 2 | 0.3 | 4 | 0.9 |  |
|  | West Asian | | 5 | 0.5 | 4 | 0.6 | 1 | 0.2 |  |
|  | Korean | | 3 | 0.3 | 2 | 0.3 | 1 | 0.2 |  |
|  | Japanese | | 4 | 0.4 | 3 | 0.5 | 1 | 0.2 |  |
|  | Other | | 28 | 2.6 | 21 | 3.4 | 7 | 1.6 |  |
|  | Prefer not to answer | | 20 | 1.9 | 7 | 1.1 | 13 | 2.9 |  |
|  |  | | *N* | % | *N* | % | *N* | % |  |
| Marital status | Common-law union | | 181 | 16.9 | 129 | 20.8^a^ | 52 | 11.6^a^ | X^2^ (6) = 30.70  *p* < .001 |
|  | Married | | 484 | 45.3 | 287 | 46.4 | 197 | 43.8 |  |
|  | Separated | | 29 | 2.7 | 19 | 3.1 | 10 | 2.2 |  |
|  | Divorced | | 61 | 5.7 | 29 | 4.7 | 32 | 7.1 |  |
|  | Single | | 285 | 26.7 | 139 | 22.5^a^ | 146 | 32.4^a^ |  |
|  | Widowed | | 25 | 2.3 | 12 | 1.9 | 13 | 2.9 |  |
|  | Other | | 4 | 0.4 | 4 | 0.6^a^ | 0 | 0.0^a^ |  |
|  |  | | *M* | *SD* | *M* | *SD* | *M* | *SD* |  |
| Number of children currently living at home | | | 0.75 | 1.00 | 0.88 | 1.03 | 0.55 | 0.93 | *t* (612) = 4.00  *p* < .001 |
|  | | | *N* | % | *N* | % | *N* | % |  |
| Diet | | Vegan | 15 | 1.4 | 13 | 2.1^a^ | 2 | 0.4^a^ | X^2^ (5) = 5.45  *p* = .363 |
|  |  | Vegetarian | 45 | 4.2 | 26 | 4.2 | 19 | 4.2 |  |
|  |  | Pescetarian | 19 | 1.8 | 12 | 1.9 | 7 | 1.6 |  |
|  | | Semi-vegetarian / Flexitarian | 100 | 9.4 | 58 | 9.4 | 42 | 9.3 |  |
|  | | Omnivore trying to reduce meat consumption | 237 | 22.2 | 135 | 21.8 | 102 | 22.7 |  |
|  | | Meat eater/Omnivore | 653 | 61.1 | 375 | 60.6 | 278 | 61.8 |  |
| Political orientation – Social issues | |  | *M* | *SD* | *M* | *SD* | *M* | *SD* |  |
|  |  |  | 3.46 | 1.76 | 3.40 | 1.80 | 3.54 | 1.71 | *t* (992) = -1.18 *p* = .237 |
| Political orientation – Economic issues | |  | *M* | *SD* | *M* | *SD* | *M* | *SD* |  |
|  |  |  | 3.85 | 1.70 | 3.84 | 1.76 | 3.87 | 1.61 | *t* (984) = -0.28 *p* = .782 |

*Notes.* Within a row, means with the same superscript differ in the Wald z-test analyses (2-tailed) comparing pet and non-pet owners at each level of the demographic variable (*p* < .05). All of the observed differences between pet and non-pet owners on the sociodemographic variables in the X^2^ and t-test analyses become non-significant when applying the second poststratification weight variable. The diet and the two political orientation variables presented in Table S1 were not included in the calculation of this second poststratification weight variable. For diet, a significant difference between pet and non-pet owners appeared in the X^2^ test when applying the second poststratification weight.

**Table S2.**

| Indirect and total effects of positive contact with pets | β | SE | *p* | [95% CI] |
| --- | --- | --- | --- | --- |
| Positive Attitudes toward Non-Pet Animals | | | | |
| Total effect of positive contact with pets | **.41** | **.03** | **.000** | **[.343, .472]** |
| Total indirect effect of positive contact with pets | **.22** | **.03** | **.000** | **[.149, .279]** |
| Positive contact with pets → Similarity with animals → Positive attitudes toward non-pet animals | .01 | .02 | .765 | [-.025, .037] |
| Positive contact with pets → Solidarity with animals → Positive attitudes toward non-pet animals | **.21** | **.04** | **.000** | **[.137, .287]** |
| Positive contact with pets → Animal pride → Positive attitudes toward non-pet animals | -.00 | .02 | .878 | [-.035, .032] |

*Indirect and Total Effects Observed When Predicting Attitudes toward Non-Pet Animals.*

*Notes*. Estimates are based on a bootstrapping procedure with 5000 bootstrap samples. The first poststratification weight was applied in these analyses.

**Table S3.**

| Indirect and total effects of positive contact with pets | β | SE | *p* | [95% CI] |
| --- | --- | --- | --- | --- |
| Positive attitudes toward outgroups | | | | |
| Total effect of positive contact with pets | **.13** | **.04** | **.001** | **[.050, .204]** |
| Total indirect effect of positive contact with pets | **.06** | **.03** | **.028** | **[.008, .118]** |
| Positive contact with pets → Similarity with animals → Positive attitudes toward outgroups | .02 | .02 | .348 | [-.018, .056] |
| Positive contact with pets → Solidarity with animals → Positive attitudes toward outgroups | .03 | .04 | .415 | [-.042, .101] |
| Positive contact with pets → Animal pride → Positive attitudes toward outgroups | .02 | .02 | .512 | [-.028, .062] |
| Positive attitudes toward ingroups | | | | |
| Total effect of positive contact with pets | **.17** | **.04** | **.000** | **[.084, .246]** |
| Total indirect effect of positive contact with pets | .03 | .03 | .327 | [-.029, .084] |
| Positive contact with pets → Similarity with animals → Positive attitudes toward ingroups | .02 | .02 | .448 | [-.021, .055] |
| Positive contact with pets → Solidarity with animals → Positive attitudes toward ingroups | .01 | .04 | .891 | [-.066, .073] |
| Positive contact with pets → Animal pride → Positive attitudes toward ingroups | .01 | .02 | .684 | [-.031, .054] |
| Social dominance orientation | | | | |
| Total effect of positive contact with pets | **-.10** | **.04** | **.017** | **[-.173, -.013]** |
| Total indirect effect of positive contact with pets | **-.07** | **.03** | **.016** | **[-.128, -.013]** |
| Positive contact with pets → Similarity with animals → Social dominance orientation | **-.04** | **.02** | **.059** | **[-.077, -.001]** |
| Positive contact with pets → Solidarity with animals → Social dominance orientation | -.07 | .04 | .082 | [-.137, .010] |
| Positive contact with pets → Animal pride → Social dominance orientation | .03 | .02 | .130 | [-.008, .071] |

*Indirect and Total Effects Observed When Predicting the Intergroup Relations Outcomes.*

*Notes*. Estimates are based on a bootstrapping procedure with 5000 bootstrap samples. The first poststratification weight was applied in these analyses.

**Table S4.**

*Indirect and Total Effects Observed When Predicting the Environmental Attitudes Outcomes.*

| Indirect and total effects of positive contact with pets | β | SE | *p* | [95% CI] |
| --- | --- | --- | --- | --- |
| Biospheric environmental concerns | | | | |
| Total effect of positive contact with pets | **.28** | **.04** | **.000** | **[.197, .352]** |
| Total indirect effect of positive contact with pets | **.22** | **.03** | **.000** | **[.162, .283]** |
| Positive contact with pets → Similarity with animals → Biospheric environmental concerns | -.01 | .02 | .386 | [-.045, .016] |
| Positive contact with pets → Solidarity with animals → Biospheric environmental concerns | **.22** | **.04** | **.000** | **[.151, .299]** |
| Positive contact with pets → Animal pride → Biospheric environmental concerns | .01 | .02 | .612 | [-.027, .046] |
| Egoistic environmental concerns | | | | |
| Total effect of positive contact with pets | -.00 | .04 | .969 | [-.082, .085] |
| Total indirect effect of positive contact with pets | **.08** | **.03** | **.016** | **[.013, .137]** |
| Positive contact with pets → Similarity with animals → Egoistic environmental concerns | .01 | .02 | .759 | [-.030, .041] |
| Positive contact with pets → Solidarity with animals → Egoistic environmental concerns | **.09** | **.04** | **.012** | **[.019, .161]** |
| Positive contact with pets → Animal pride → Egoistic environmental concerns | -.02 | .02 | .322 | [-.058, .020] |
| Altruistic environmental concerns | | | | |
| Total effect of positive contact with pets | .06 | .04 | .187 | [-.025, .142] |
| Total indirect effect of positive contact with pets | **.09** | **.03** | **.001** | **[.037, .148]** |
| Positive contact with pets → Similarity with animals → Altruistic environmental concerns | .01 | .02 | .662 | [-.025, .042] |
| Positive contact with pets → Solidarity with animals → Altruistic environmental concerns | **.09** | **.03** | **.004** | **[.033, .160]** |
| Positive contact with pets → Animal pride → Altruistic environmental concerns | -.01 | .02 | .540 | [-.049, .024] |
| Inclusion of nature in the self | | | | |
| Total effect of positive contact with pets | **.15** | **.04** | **.000** | **[.067, .232]** |
| Total indirect effect of positive contact with pets | **.07** | **.03** | **.026** | **[.010, .135]** |
| Positive contact with pets → Similarity with animals → Inclusion of nature in the self | .02 | .02 | .417 | [-.019, .052] |
| Positive contact with pets → Solidarity with animals → Inclusion of nature in the self | .07 | .04 | .099 | [-.011, .146] |
| Positive contact with pets → Animal pride → Inclusion of nature in the self | -.01 | .02 | .683 | [-.051, .032] |
| Human-environment interdependence beliefs | | | | |
| Total effect of positive contact with pets | **.20** | **.04** | **.000** | **[.124, .273]** |
| Total indirect effect of positive contact with pets | **.14** | **.03** | **.000** | **[.090, .202]** |
| Positive contact with pets → Similarity with animals → Human-environment interdependence beliefs | .00 | .02 | .795 | [-.028, .037] |
| Positive contact with pets → Solidarity with animals → Human-environment interdependence beliefs | **.14** | **.03** | **.000** | **[.072, .206]** |
| Positive contact with pets → Animal pride → Human-environment interdependence beliefs | .00 | .02 | .932 | [-.034, .039] |

*Notes*. Estimates are based on a bootstrapping procedure with 5000 bootstrap samples. The first poststratification weight was applied in these analyses.

**Table S5.**

*Indirect and Total Effects Observed When Predicting the Meat Consumption Outcomes.*

| Indirect and total effects of positive contact with pets | β | SE | *p* | [95% CI] |
| --- | --- | --- | --- | --- |
| Number of weekly meat portions | | | | |
| Total effect of positive contact with pets | -.04 | .04 | .304 | [-.112, .035] |
| Total indirect effect of positive contact with pets | -.03 | .03 | .267 | [-.089, .019] |
| Positive contact with pets → Similarity with animals → Number of weekly meat portions | .01 | .02 | .468 | [-.024, .050] |
| Positive contact with pets → Solidarity with animals → Number of weekly meat portions | **-.08** | **.03** | **.016** | **[-.142, -.019]** |
| Positive contact with pets → Animal pride → Number of weekly meat portions | .03 | .02 | .116 | [-.006, .074] |
| Diet | | | | |
| Total effect of positive contact with pets | -.01 | .04 | .741 | [-.093, .065] |
| Total indirect effect of positive contact with pets | **-.09** | **.04** | **.008** | **[-.162, -.024]** |
| Positive contact with pets → Similarity with animals → Diet | .02 | .02 | .360 | [-.017, .058] |
| Positive contact with pets → Solidarity with animals → Diet | **-.10** | **.04** | **.021** | **[-.189, -.017]** |
| Positive contact with pets → Animal pride → Diet | -.01 | .02 | .693 | [-.058, .034] |
| Rationalization of meat consumption | | | | |
| Total effect of positive contact with pets | -.05 | .04 | .226 | [-.133, .034] |
| Total indirect effect of positive contact with pets | -.04 | .03 | .228 | [-.110, .023] |
| Positive contact with pets → Similarity with animals → Rationalization of meat consumption | -.01 | .02 | .467 | [-.054, .024] |
| Positive contact with pets → Solidarity with animals → Rationalization of meat consumption | .00 | .04 | .993 | [-.084, .078] |
| Positive contact with pets → Animal pride → Rationalization of meat consumption | -.03 | .02 | .209 | [-.073, .013] |

*Notes*. Estimates are based on a bootstrapping procedure with 5000 bootstrap samples. The first poststratification weight was applied in these analyses.

**Table S6.**

|  | *Pet Owners*  *(n=619)* | | *Non-Pet Owners*  *(n=450)* | | *Pet Ownership Effect* | | | |
| --- | --- | --- | --- | --- | --- | --- | --- | --- |
|  | *M* | *SD* | *M* | *SD* | *df* | *t* | Cohen’s d | *p* |
| Conflict with being an animal | 4.48 | 2.13 | 4.83 | 2.12 | 1067 | -2.66 | 0.17 | .008 |
| Superiority of humans relative to animals | 4.57 | 1.19 | 4.94 | 1.12 | 1067 | -5.19 | 0.32 | <.001 |
| Similarity of animals to humans | 4.22 | 1.19 | 3.82 | 1.32 | 904 | 5.05 | 0.32 | <.001 |
| Anthropocentrism | 2.59 | 0.98 | 2.92 | 0.95 | 1060 | -5.56 | 0.35 | <.001 |
| Positive attitudes toward pets | 5.36 | 0.86 | 4.69 | 1.06 | 845 | 11.18 | 0.72 | <.001 |
| Positive attitudes toward wild animals | 5.23 | 1.02 | 4.71 | 1.10 | 1067 | 7.97 | 0.49 | <.001 |
| Positive attitudes toward pests | 3.53 | 1.41 | 2.86 | 1.14 | 1056 | 8.64 | 0.52 | <.001 |
| Positive attitudes toward farm animals | 5.22 | 1.03 | 4.71 | 1.01 | 1067 | 7.98 | 0.49 | <.001 |

*Results of the Independent-Sample T-Tests Comparing Pet and Non-Pet Owners on the Additional Perceptions of Animals and of Humans Variables*

*Notes.* Reliabilities for these measures were satisfactory: conflict with being an animal (α=.85), superiority of humans relative to animals (α=.76), similarity of animals to humans is a one-item measure, anthropocentrism (α=.86), positive attitudes toward pets (α=.62), positive attitudes toward wild animals (α=.82), positive attitudes toward pests (α=.80), and positive attitudes toward farm animals (α=.88). The second poststratification weight was applied in these analyses.

**Table S7.**

*Multiple Regressions Predicting Positive Contact with Pets and the Additional Perceptions of Animals and of Humans Variables.*

|  | Dimensions of Identification | | |  |
| --- | --- | --- | --- | --- |
|  | Human-Animal Similarity | Solidarity with Animals | Animal Pride |  |
|  | *β* [95% CI] | *β* [95% CI] | *β* [95% CI] | *R^2^* |
| Positive contact with pets | -.06 [-.150,.018] | .60^***^ [.601,.752] | -.01 [-.077,.067] | .32^***^ |
| Conflict with being an animal | .08 [-.014, .278] | -.05 [-.211, .050] | -.12^*^ [-.287, -.037] | .01^**^ |
| Superiority of humans relative to animals | -.11^*^ [-.177, -.021] | -.15^***^ [-.200, .-060] | -.08 [-.130, .004] | .09^***^ |
| Similarity of animals to humans | .32^***^ [.234, .393] | .06 [-.017, .125] | .08 [-.003, .133] | .18^***^ |
| Anthropocentrism | -.09^*^ [-.127,.-.001] | -.37^***^ [-.334, -.221] | .07 [-.012,.096] | .15^***^ |
| Positive attitudes toward pets | .01 [-.056, .070] | .50^***^ [.342, .454] | .01 [-.050, .058] | .26^***^ |
| Positive attitudes toward wild animals | .03 [-.043, .092] | .46^***^ [.319, .439] | -.05 [-.093, .022] | .20^***^ |
| Positive attitudes toward pests | -.02 [-.104, .065] | .37^***^ [.299, .450] | .08 [-.006, .139] | .17^***^ |
| Positive attitudes toward farm animals | .00 [-.063, .070] | .46^***^ [.317, .436] | -.07 [-.105, .009] | .18^***^ |

*Notes.* **p* < 0.05; ***p* < 0.01; ****p* < 0.001. All tolerance values were above 0.42 and all variance inflation factors values (VIF) were equal to or less than 2.40, indicating that multicollinearity was not a problem in these analyses. The first poststratification weight was applied in these analyses.

**Table S8.**

*Direct Effects Observed in the Mediation Models Predicting the Additional Perceptions of Animals and of Humans Variables.*

|  | Predictors | | | | | | | | | | | | |
| --- | --- | --- | --- | --- | --- | --- | --- | --- | --- | --- | --- | --- | --- |
|  | Positive contact with pets | | | Human-Animal Similarity | | | Solidarity with Animals | | | Animal Pride | | | |
|  | β [95% CI] | SE | *p* | β [95% CI] | SE | *p* | β [95% CI] | SE | *p* | β [95% CI] | SE | *p* |  |
| Human-Animal Similarity | **.30 [.220, .380]** | **.04** | **.000** |  |  |  |  |  |  |  |  |  |  |
| Solidarity with Animals | **.56 [.497, .622]** | **.03** | **.000** |  |  |  |  |  |  |  |  |  |  |
| Animal Pride | **.34 [.258, .412]** | **.04** | **.000** |  |  |  |  |  |  |  |  |  |  |
| Conflict with being an animal | -.03 [-.128, .085] | .05 | .636 | .08 [-.036, .192] | .06 | .167 | -.03 [-.167, .102] | .07 | .617 | -.12 [-.257, .012] | .07 | .080 |  |
| Superiority of humans relative to animals | **-.10 [-196., -.010]** | **.05** | **.034** | -.12 [-.232, .000] | .06 | .052 | -.09 [-.203, .040] | .06 | .179 | -.08 [-.204, .037] | .06 | .174 |  |
| Similarity of animals to humans | .07 [-.020, .165] | .05 | .121 | **.33 [.217, .435]** | **.06** | **.000** | .01 [-.103, .134] | .06 | .830 | .08 [-.046, .200] | .06 | .200 |  |
| Anthropocentrism | **-.19 [-.279, -.091]** | **.05** | **.000** | -.10 [-.200, .013] | .05 | .077 | **-.26 [-.374, -.138]** | **.06** | **.000** | .07 [-.043, .165] | .05 | .219 |  |
| Positive attitudes toward pets | **.33 [.249, .415]** | **.04** | **.000** | .03 [-.068, .121] | .05 | .557 | **.30 [.190, .393]** | **.05** | **.000** | .01 [-.092, .118] | .05 | .888 |  |
| Positive attitudes toward wild animals | **.19 [.099, .271]** | **.04** | **.000** | .04 [-.056, .145] | .05 | .436 | **.35 [.245, .445]** | **.05** | **.000** | -.05 [-.146, .050] | .05 | .313 |  |
| Positive attitudes toward pests | **.14 [.049, .238]** | **.05** | **.004** | -.01 [-.116, .095] | .05 | .840 | **.29 [.148, .398]** | **.06** | **.000** | .08 [-.035, .189] | .06 | .171 |  |
| Positive attitudes toward farm animals | **.19 [.097, .282]** | **.05** | **.000** | .02 [-.084, .117] | .05 | .758 | **.35 [.222, .461]** | **.06** | **.000** | -.07 [-.169, .034] | .05 | .180 |  |

*Notes*. Estimates are based on a bootstrapping procedure with 5000 bootstrap samples. The first poststratification weight was applied in these analyses.

**Table S9.**

| Indirect and total effects of positive contact with pets | *β* | SE | *p* | [95% CI] |
| --- | --- | --- | --- | --- |
| Conflict with being an animal | | | | |
| Total effect of positive contact with pets | -.06 | .05 | .179 | [-.149, .029] |
| Total indirect effect of positive contact with pets | -.04 | .03 | .234 | [-.096, .022] |
| Positive contact with pets → Similarity with animals → Conflict with being an animal | .02 | .02 | .167 | [-.009, .060] |
| Positive contact with pets → Solidarity with animals → Conflict with being an animal | -.02 | .04 | .618 | [-.095, .057] |
| Positive contact with pets → Animal pride → Conflict with being an animal | -.04 | .02 | .083 | [-.089, .004] |
| Superiority of humans relative to animals | | | | |
| Total effect of positive contact with pets | **-.21** | **.04** | **.000** | **[-.293, -.124]** |
| Total indirect effect of positive contact with pets | **-.11** | **.03** | **.000** | **[-.168, -.049]** |
| Positive contact with pets → Similarity with animals → Superiority of humans relative to animals | **-.04** | **.02** | **.060** | **[-.075, -.001]** |
| Positive contact with pets → Solidarity with animals → Superiority of humans relative to animals | -.05 | .04 | .181 | [-.116, .022] |
| Positive contact with pets → Animal pride → Superiority of humans relative to animals | -.03 | .02 | .184 | [-.072, .012] |
| Similarity of animals to humans | | | | |
| Total effect of positive contact with pets | **.21** | **.04** | **.000** | **[.118, .293]** |
| Total indirect effect of positive contact with pets | **.13** | **.03** | **.000** | **[.067, .198]** |
| Positive contact with pets → Similarity with animals → Similarity of animals to humans | **.10** | **.02** | **.000** | **[.063, .142]** |
| Positive contact with pets → Solidarity with animals → Similarity of animals to humans | .01 | .03 | .831 | [-.059, .077] |
| Positive contact with pets → Animal pride → Similarity of animals to humans | .03 | .02 | .219 | [-.015, .072] |
| Anthropocentrism | | | | |
| Total effect of positive contact with pets | **-.34** | **.04** | **.000** | **[-.407, -.262]** |
| Total indirect effect of positive contact with pets | **-.15** | **.03** | **.000** | **[-.214, -.097]** |
| Positive contact with pets → Similarity with animals → Anthropocentrism | -.03 | .02 | .087 | [-.066, .003] |
| Positive contact with pets → Solidarity with animals → Anthropocentrism | **-.15** | **.04** | **.000** | **[-.221, -.076]** |
| Positive contact with pets → Animal pride → Anthropocentrism | .02 | .02 | .224 | [-.013, .058] |

*Indirect and Total Effects Observed When Predicting the Additional Perceptions of Animals and of Humans Variables.*

*Notes*. Estimates are based on a bootstrapping procedure with 5000 bootstrap samples. The first poststratification weight was applied in these analyses.

**Table S10.**

| Indirect and total effects of positive contact with pets | β | SE | *p* | [95% CI] |
| --- | --- | --- | --- | --- |
| Positive attitudes toward pets | | | | |
| Total effect of positive contact with pets | **.51** | **.03** | **.000** | **[.448, .570]** |
| Total indirect effect of positive contact with pets | **.18** | **.03** | **.000** | **[.125, .236]** |
| Positive contact with pets → Similarity with animals → Positive attitudes toward pets | .01 | .02 | .565 | [-.020, .040] |
| Positive contact with pets → Solidarity with animals → Positive attitudes toward pets | **.17** | **.03** | **.000** | **[.107, .229]** |
| Positive contact with pets → Animal pride → Positive attitudes toward pets | .00 | .02 | .888 | [-.031, .040] |
| Positive attitudes toward wild animals | | | | |
| Total effect of positive contact with pets | **.38** | **.04** | **.000** | **[.301, .447]** |
| Total indirect effect of positive contact with pets | **.19** | **.03** | **.000** | **[.136, .251]** |
| Positive contact with pets → Similarity with animals → Positive attitudes toward wild animals | .01 | .02 | .446 | [-.017, .046] |
| Positive contact with pets → Solidarity with animals → Positive attitudes toward wild animals | **.20** | **.03** | **.000** | **[.136, .262]** |
| Positive contact with pets → Animal pride → Positive attitudes toward wild animals | -.02 | .02 | .313 | [-.050, .016] |
| Positive attitudes toward pests | | | | |
| Total effect of positive contact with pets | **.32** | **.04** | **.000** | **[.251, .388]** |
| Total indirect effect of positive contact with pets | **.18** | **.03** | **.000** | **[.121, .245]** |
| Positive contact with pets → Similarity with animals → Positive attitudes toward pests | -.00 | .02 | .842 | [-.036, .029] |
| Positive contact with pets → Solidarity with animals → Positive attitudes toward pests | **.16** | **.04** | **.000** | **[.082, .233]** |
| Positive contact with pets → Animal pride → Positive attitudes toward pests | .03 | .02 | .183 | [-.011, .067] |
| Positive attitudes toward farm animals | | | | |
| Total effect of positive contact with pets | **.37** | **.03** | **.000** | **[.301, .435]** |
| Total indirect effect of positive contact with pets | **.18** | **.03** | **.000** | **[.116, .243]** |
| Positive contact with pets → Similarity with animals → Positive attitudes toward farm animals | .01 | .02 | .762 | [-.025, .039] |
| Positive contact with pets → Solidarity with animals → Positive attitudes toward farm animals | **.20** | **.04** | **.000** | **[.124, .273]** |
| Positive contact with pets → Animal pride → Positive attitudes toward farm animals | -.02 | .02 | .180 | [-.058, .011] |

*Indirect and Total Effects Observed When Predicting the Additional Perceptions of Animals and of Humans Variables.*

*Notes*. Estimates are based on a bootstrapping procedure with 5000 bootstrap samples. The first poststratification weight was applied in these analyses.

**Questionnaire**

Would you prefer to complete the survey in English or French?

Préféreriez-vous répondre à ce questionnaire en anglais ou en français?

- English / Anglais
- Français / French

**PERCEPTIONS OF ANIMALS AND OF HUMANS: IDENTIFICATION WITH ANIMALS, SOCIAL ATTITUDES, AND CURRENT ISSUES**

INFORMATION AND CONSENT FORM

INTRODUCTION:

You are invited to take part in a research project that investigates peoples’ perceptions of animals and of humans, and attitudes towards different social issues. We also aim to test the associations that may exist between the different ways people feel psychologically connected to animals, their perceptions of animals, humans, and nature, as well as their feelings and behaviours towards animals and towards different human social groups. Before accepting to participate in this study, please take the time required to read the following information. If you have any questions about the information contained in this consent form, please do not hesitate to contact us.

IDENTIFICATION:

(Information of this section has been removed to ensure double-blind review)

FUNDING:

This study is funded by the Social Sciences and Humanities Research Council of Canada (SSHRC).

PROCEDURES OR TASKS REQUESTED FROM PARTICIPANTS:

Your participation involves completing a 30-minute online questionnaire. You will be invited to answer different questions concerning the way you may identify with animals, your perceptions of animals, humans, and nature, as well as your feelings and behaviours towards animals and towards different human social groups. More specifically, the questionnaire involves completing scales that measure: your connection to animals in general; your contacts with pets; your attitudes towards different types of animals; your perceptions of the status of animals compared to humans; your beliefs about the environment and nature, and humans’ place within it; your feelings towards different social groups; your perceptions of social hierarchy; and your opinions about meat consumption. Please complete this questionnaire on a computer. This ensures the quality of the information.

BENEFITS AND RISKS:

Your participation will contribute to a better understanding of the ways we can feel connected to and identify with animals, and what are the implications of this psychological connection, for animals but also for other humans. This represents a major advancement in the field of social psychology, since to date, only a few studies have examined this type of psychological connection as well as its consequences for animals and for humans. There is no risk of significant discomfort associated with your participation. Please keep in mind when completing the questionnaire that there are no right or wrong answers; we are interested in how you actually think and feel. However, you must be aware that some questions may rekindle some unpleasant emotions. Please keep in mind that you remain free not to answer a question that may cause you discomfort without having to justify yourself. You can also stop participating in the study at any time. Support resources are also provided at the end of the study.

ANONYMITY AND CONFIDENTIALITY:

All information collected in the questionnaire will remain confidential. The data that will be gathered for this study will be kept in a secured database and the online questionnaire is hosted on a secure server. Only (information removed to ensure double-blind review) and the research assistant(s) involved in this study will have access to the data. Data will be analyzed globally and it will not be possible to identify a specific participant. The data gathered for this study will be published in scientific journals, for scientific and educational purposes only. In keeping with best practices of open and transparent scientific research, we ask your permission to share some of the data you have provided with other researchers. Specifically, the computerized databases will be made available online to other researchers, but only for data verification purposes. However, these databases will not contain any identifying information (e.g., email).

VOLUNTARY PARTICIPATION:

Your participation in this study is entirely voluntary. This means that you agree to participate in the study without any constraint or external pressure. Moreover, you are free to terminate your participation at any time during the study, without prejudice of any nature and without having to justify yourself. Your agreement to participate in this study also means that you agree that the research team may use the information collected for scientific and educational purposes (e.g., articles, theses of student team members, presentations in conferences, and scientific papers) on the condition that no information that identifies you is publicly disclosed unless your explicit consent is obtained. Please note that we will not be able to destroy the questionnaire after submission since all data are anonymous.

CLAUSE OF LIABILITY:

By agreeing to participate in this project, you do not waive any of your rights or release the researchers, the sponsor, or the institutions involved from their legal and professional obligations. At the end of this project, we would like to retain your data for an unlimited period of time to be able to conduct other research projects. This involves analyzing your current data according to new research hypotheses. The ethical rules of this project apply to the long-term preservation of your data.

QUESTIONS ABOUT THE PROJECT OR YOUR RIGHTS?

(Information of this section has been removed to ensure double-blind review)

CONSENT:

Hereby:

a) I have read this information and consent form;

b) I voluntarily consent to participate in this research project;

c) I understand the goals of the project and what my participation implies;

d) I confirm that I have had sufficient time to consider my decision to participate in this study;

e) I also recognize that the project manager and the research team have answered my questions satisfactorily; and

f) I understand that my participation in this research is completely voluntary and that I can withdraw from the study at any time without any penalties and without having to justify myself.

If you agree to participate in this study, please indicate your consent by choosing the "Yes, I consent to participate" answer below, which will direct you to the online questionnaire.

- Yes, I consent to participate.
- No

**[age]** **What is your age?**

[r1] Under 18 years old

[r2] 18 to 21 years old

[r3] 22 to 24 years old

[r4] 25 to 29 years old

[r5] 30 to 34 years old

[r6] 35 to 39 years old

[r7] 40 to 44 years old

[r8] 45 to 49 years old

[r9] 50 to 54 years old

[r10] 55 to 59 years old

[r11] 60 to 64 years old

[r12] 65 to 69 years old

[r13] 70 to 74 years old

[r14] 75 or older

[gender] What is your gender?

[r1] Male

[r2] Female

[r96] Or please specify your gender

[ethn] What is your ethnicity?

[r1] White

[r2] South Asian (e.g., East Indian, Pakistani, Sri Lankan)

[r3] Chinese

[r4] Black

[r5] Filipino

[r6] Arab

[r7] Latin American

[r8] Southeast Asian (e.g., Vietnamese, Cambodian, Laotian, Thai)

[r9] West Asian (e.g., Iranian, Afghan)

[r10] Korean

[r11] Japanese

[r96] Other group, specify:

[r99] I prefer not to answer

[AREA] In what area do you live?

[r1] City

[r2] Suburb

[r3] Countryside

[DWELLING] What type of dwelling do you live in?

[r1] Apartment/Condo

[r2] House

[r96] Other, please specify:

[MartialStatus] What is your marital status?

[r1] Common-law union

[r2] Married

[r3] Separated

[r4] Divorced

[r5] Single

[r6] Widowed

[r7] Single parent

[r96] Other, please specify:

[childWithYou] How many of your children who are younger than 18 years old currently live with you?:

[workArrang] Please indicate your current work arrangements:

[r1] Full-time

[r2] Part-time

[r3] Self-employed

[r4] Student

[r5] Homemaker

[r6] Unemployed

[r7] Retired

[EDUC] What is the highest degree/qualification that you have earned?

[r1] Primary school diploma

[r2] High school diploma

[r3] Diploma of Collegial studies (e.g., College, CÉGEP, Technical training, certificates)

[r4] University: Certificates and diplomas

[r5] University: Bachelor’s degree

[r6] University: Master’s degree

[r7] University: Doctoral degree

[r96] Other, please specify:

[INCOME] Among the following categories, which one best reflects the total income, before taxes, of all members of your household for the year 2021?

[r1] Less than $20,000

[r2] $20, 000 to $39, 999

[r3] $40, 000 to $59, 999

[r4] $60, 000 to $79, 999

[r5] $80, 000 to $99, 999

[r6] $100, 000 to $119, 999

[r7] $120, 000 to $139, 999

[r8] $140, 000 to $159, 999

[r9] $160, 000 to $179, 999

[r10] $180, 000 to $199, 999

[r11] Over $200, 000

[r99] I prefer not to answer

[polEco] Please indicate your political beliefs from left/liberal to right/conservative on issues of the economy (e.g., social welfare, government spending, tax cuts):

[r1] Left/Liberal (1)

[r2] (2)

[r3] (3)

[r4] (4)

[r5] (5)

[r6] (6)

[r7] Right/Conservative (7)

[r99] I prefer not to answer (99)

[polSoc] Please indicate your political beliefs from left/liberal to right/conservative on social issues (e.g., immigration, homosexual marriage, abortion):

[r1] Left/Liberal (1)

[r2] (2)

[r3] (3)

[r4] (4)

[r5] (5)

[r6] (6)

[r7] Right/Conservative (7)

[r99] I prefer not to answer (99)

[DIET] Which of the following best describes your diet?

[r1] Vegan (no animal products consumed)

[r2] Vegetarian (includes eating some animal-derived foods like dairy products and eggs)

[r3] Pescatarian (includes eating fish and shellfish but no meat)

[r4] Semi-vegetarian / Flexitarian (involves occasionally eating meat and animal products)

[r5] Omnivore trying to reduce meat consumption

[r6] Meat eater / Omnivore (no restriction in eating animal products)

[portions_meat] How many portions of meat do you eat per week (on average)?:

[petOwn] Do you have one or more pet(s) currently?

[r1] Yes

[r2] No

[id_simi] [id_soli] [id_sati] Social identification with animals (IWAM; Amiot et al., 2020)

The term "animal" is often reserved for non-human animals, but in fact it is a category that both humans and other animals belong to. Keeping this broader and more inclusive definition of "animal" in mind and using the scale below, please respond to the following questions.

*Rating scale:*

1. Not agree at all
2. Agree very slightly
3. Slightly agree
4. Moderately agree
5. Agree
6. Strongly agree
7. Very strongly agree

*Items:*

[id_simi1] Animals are a lot alike in many respects.

[id_soli1] I feel committed to animals.

[id_sati1] I am proud to be an animal.

[id_soli2] I feel good about animals.

[id_simi2] Animals, including human animals, are very similar to each other.

[id_soli3] I often exhibit my positive feelings about animals.

[id_simi3] I have a lot in common with the average animal.

[id_sati2] It is pleasant to be an animal.

[id_soli4] I feel solidarity with animals.

[id_sati3] It is good to be an animal.

[id_soli5] I would take a substantial risk to rescue an animal in trouble.

[id_sati4] Generally, I feel good when I think about myself as an animal.

[id_simi4] Animals, including human animals, have a lot in common with each other.

[id_sati5] Being an animal gives me a good feeling.

[id_sati6] I am glad to be an animal.

[**Tens**] **Conflict with being an animal (items adapted from Berndsen and van der Pligt, 2004)**

Using the following scales, please indicate your attitudes toward being an animal:

[Tens1] *Responses were recorded on a scale from 1 (I feel no* ***ambivalence*** *at all) to 4 (I feel maximum* ***ambivalence****)*

[Tens2] *Responses were recorded on a scale from 1 (I feel no* ***tension*** *at all) to 4 (I feel maximum* ***tension****)*

[Tens3] *Responses were recorded on a scale from 1 (I feel no* ***conflict*** *at all) to 4 (I feel maximum* ***conflict****)*

[contact] Positive contact with pets (three items from Auger and Amiot (2017, 2019); two new items)

We now ask you to refer to your contact with pets. Using the scale below, please evaluate the frequency at which…

*Rating scale:*

1. Never
2. Rarely
3. Sometimes
4. Often
5. Very often
6. Always

*Items:*

[contact1] You are in contact with pets.

[contact2] You have mutual interactions with pets (play, pet, communicate with them, etc.).

[contact3] Your contact with pets provides you with positive emotions (joy, enthusiasm, feeling of belongingness, etc.).

[contact4] You spend time with pets.

[contact5] You do activities with pets.

[non_pet] [pet] [pest] [farm] [pest] Positive attitudes toward different species of animals

Please indicate to what extent you like or dislike the following types of animals:

*Rating scale:*

1. Strongly Dislike
2. Dislike
3. Somewhat Dislike
4. Neither Like nor Dislike
5. Somewhat Like
6. Like
7. Strongly Like

*Items:*

[type1] Moose

[type2] Rats

[type3] Sheep

[type4] Guinea pigs

[type5] Cats

[type6] Beavers

[type7] Pigeons

[type8] Blue jays

[type9] Serpents

[type10] Buggies

[type11] Hare

[type12] Dogs

[type13] Pigs

[type14] Cows

[type15] Hens

[type16] Skunks

**[status_diff_no1]** **Superiority of humans relative to animals (Amiot, Sukhanova, Greenaway, & Bastian, 2017)**

[status_diff2] Are animals inferior to humans?

*Rating scale:*

1. Animals are very much inferior to humans
2. Animals are the equivalent of humans in terms of superiority
3. Animals are very much superior to humans

[status_diff3] Are humans superior to animals?

*Rating scale:*

1. Humans are very much inferior to animals
2. Humans are the equivalent of animals in terms of superiority
3. Humans are very much superior to animals

[status_diff4] How exceptional are humans compared to animals?

*Rating scale:*

1. Humans are not exceptional at all
2. Humans are moderately exceptional compared to animals
3. Humans are very much exceptional compared to animals

[status_diff5] Similarity of animals to humans (Amiot, Sukhanova, Greenaway, & Bastian, 2017)

To what extent are animals similar to humans?

*Rating scale:*

1. Not at all similar
2. Moderately similar
3. Completely similar

[anthro] Anthropocentrism (subscale from Lund, Kondrup & Sandøe, 2019)

Please indicate the extent to which you agree or disagree with each statement using the following rating scale.

*Rating scale:*

1. Totally disagree
2. Disagree
3. Neither agree nor disagree
4. Agree
5. Totally agree

*Items:*

[anthro1] We have the right to use animals because humans are intellectually superior to animals.

[anthro2] Human interests are more important than those of animals

[anthro3] We must prioritize humans over animals.

[bio] [alt] [ego] Biospheric, Egoistic, and Altruistic Environmental concerns (Schultz, 2000)

People around the world are generally concerned about environmental problems because of the consequences that result from harming nature. However, people differ in the consequences that concern them the most. Please rate each of the following items from 1 (not important) to 7 (supreme importance) in response to the question: I am concerned about environmental problems because of the consequences for ____:

*Responses were recorded on a scale from 1 (Not important) to 4 (Supreme importance)*

[bio1] Whales

[bio2] Animals

[alt1] People in my community

[bio3] Birds

[bio4] Marine life

[ego1] My health

[bio6] Plants

[ego3] My lifestyle

[ego4] My future

[bio5] Trees

[alt2] Humanity

[ego5] Me

[alt3] Children

[alt4] Future generations

[ego2] My prosperity

**[ins]** **Inclusion of Nature in the Self (Schultz, 2001)**

Please select the picture that best describes your relationship with the natural environment. How interconnected are you with nature?

Nature

Self

Nature

Self

Nature

Self

(A) (B) (C)

Self

Nature

Nature

Self

Nature

Self

Nature

Self

(D) (E) (F) (G)

[nhip] Human-environment interdependence beliefs: New Human Interdependence Paradigm (NHIP; Corral-Verdugo et al., 2008)

Please indicate the extent to which you agree or disagree with each statement using the following rating scale.

*Responses were recorded on a scale from 1 (Strongly disagree) to 5 (Strongly agree)*

[nhip1] Human beings can progress only by conserving nature’s resources.

[nhip2] Human beings can enjoy nature only if they make wise use of its resources.

[nhip3] Human progress can be achieved only by maintaining ecological balance.

[nhip4] Preserving nature now means ensuring the future of human beings.

[nhip5] We must reduce our consumption levels to ensure well-being of the present and future generations.

**[thermExo] [thermEndo] Positive attitudes toward outgroups and Positive attitudes toward ingroups**

Look at this thermometer:

**Negative or cold feelings**

**Positive feelings**

*Responses were recorded on a scale from 1 (Negative or cold feelings) to 100 (Positive feelings)*

Using this thermometer as a guide, please indicate how you feel toward the members of different groups listed below.

Indicate your feelings toward each of these groups by inserting a number between 0 and 100 in the spaces below. You can use any number between 0 and 100. Please use higher numbers to indicate positive feelings and smaller numbers to indicate negative or cold feelings.

[therm1] People with different religious beliefs

[therm2] Members of your family

[therm3] People from other ethnic communities

[therm5] Citizens of your country

[therm7] Supporters of opposing political party

[therm8] Citizens of other countries

**[sdo] Social dominance orientation: SDO_7(s)_ scale (Ho et al., 2015)**

Show how much you favour or oppose each idea below by selecting a number from 1 (strongly oppose) to 7 (strongly favour) on the scale below. You can work quickly; your first feeling is generally best.

*Rating scale:*

1. Strongly Oppose
2. Somewhat Oppose
3. Slightly Oppose
4. Neutral
5. Slightly Favour
6. Somewhat Favour
7. Strongly Favour

*Items:*

[sdo1] Some groups of people are simply inferior to other groups.

[sdo2] We should do what we can to equalize conditions for different groups.

[sdo3] We should work to give all groups an equal chance to succeed.

[sdo4] It is unjust to try to make groups equal.

[sdo5] No one group should dominate in society.

[sdo6] Group equality should not be our primary goal.

[sdo7] Groups at the bottom are just as deserving as groups at the top.

[sdo8] An ideal society requires some groups to be on top and others to be on the bottom.

**[fourNs] Rationalization of meat consumption: 4N Scale (Piazza et al., 2015)**

Please indicate the extent to which you agree or disagree with each statement using the following rating scale.

*Rating scale:*

1. Completely disagree
2. Somewhat Oppose
3. Slightly Oppose
4. Neither agree nor disagree
5. Slightly Favour
6. Somewhat Favour
7. Completely agree

*Items:*

[4ni1] Meals without meat would just be bland and boring.

[4na1] Our human ancestors ate meat all the time.

[4na2] It is only natural to eat meat.

[4no1] Not eating meat is socially unacceptable.

[4na3] Human beings naturally crave meat.

[4na4] It is unnatural to eat an all plant-based diet.

[4no2] It is normal to eat meat.

[4ne1] A healthy diet requires at least some meat.

[4ni2] Meat is delicious.

[4ne2] Human beings need to eat meat.

[4ni3] Meat adds so much flavour to a meal it does not make sense to leave it out.

[4ne3] It is necessary to eat meat in order to be healthy.

[4ne4] You cannot get all the protein, vitamins, and minerals you need on an all plant-based diet.

[4no3] Most people I know eat meat.

[4no4] It is abnormal for humans not to eat meat.

[4ni4] The best tasting food is normally a meat based dish (e.g., steak, chicken breast, grilled fish).
